# Utilization of multiwalled boron nitride nanotubes for the reinforcement of lightweight aluminum ribbons

**DOI:** 10.1186/1556-276X-8-3

**Published:** 2013-01-02

**Authors:** Maho Yamaguchi, Amir Pakdel, Chunyi Zhi, Yoshio Bando, Dai-Ming Tang, Konstantin Faerstein, Dmitry Shtansky, Dmitri Golberg

**Affiliations:** 1 World Premier International (WPI) Center for Materials Nanoarchitectonics (MANA), National Institute for Materials Science (NIMS), Namiki 1-1, Tsukuba, Ibaraki, 305-0044, Japan; 2 Graduate School of Pure and Applied Science, University of Tsukuba, Tennodai 1-1-1, Tsukuba, 305-8577, Japan; 3 Department of Physics and Materials Science, City University of Hong Kong, Tat Chee Avenue, Kowloon, Hong Kong, 8523, China; 4 Shenyang National Laboratory for Materials Science, Institute of Metal Research, Chinese Academy of Sciences, 72 Wenhua Road, Shenyang, 110016, China; 5 National University of Science and Technology “MISIS”, Leninsky Avenue 4, Moscow, 119049, Russian Federation

**Keywords:** Aluminum, Boron nitride, Composite, Nanotubes, Melt spinning

## Abstract

Multiwalled boron nitride nanotubes (BNNTs) have very attractive mechanical and thermal properties, e.g., elasticity, tensile strength, and high resistance to oxidation, and may be considered as ideal reinforcing agents in lightweight metal matrix composites. Herein, for the first time, Al-BNNT ribbons with various BNNT contents (up to 3 wt.%) were fabricated via melt spinning in an argon atmosphere. BNNTs were randomly dispersed within a microcrystalline Al matrix under ribbon casting and led to more than doubling of room-temperature ultimate tensile strength of the composites compared to pure Al ribbons produced at the similar conditions.

## Background

The importance of making lightweight but high-strength structural materials has long been recognized [1]. These days, metal matrix composites (MMCs) based on lightweight metals are extensively used in aerospace and automotive industries. Over the last decade, much research has been carried out in the field of standard carbon nanotube (CNT)-MMCs [1]. Among common aircraft materials, an Al matrix has been the most popular one for the CNT-MMC studies. There has been a variety of methods such as powder metallurgy or melting and solidification processes which have been tried to fabricate CNT-MMCs. According to a review by Bakshi et al. [1], most of Al-CNT composites were prepared by a powder metallurgy route; however, these revealed several and rather severe technological drawbacks. For example, formation of aluminum carbide (Al_4_C_3_) in an Al-CNT matrix took place, and according to some reports, this effect reduced the composite mechanical strength [2]; the others, by contrast, mentioned that some amount of Al_4_C_3_ had helped in the effective load transfer and pinning of CNTs to the matrix [3]. Another problem is the large surface area of CNTs which led to the formation of nanotube clusters due to van der Waals forces, CNT bundling and entanglement within the matrix, and related difficulties in their uniform dispersion in Al. This, in turn, created internal stresses and/or microvoids and resulted in an insurmountable cracking at composite loading [4-6]. Also, in air, the CNTs typically start to burn at around 500°C to 600°C, thus restricting medium- and high-temperature CNT-MMC applications.

Boron nitride nanotubes (BNNTs) are another type of nanotubes with a very similar crystal structure to that of CNTs in which alternating B and N atoms substitute for C atoms in a honeycomb lattice. They exhibit many exciting properties, particularly valuable for structural and composite applications. First of all, BNNTs are chemically and thermally much more robust compared to CNTs. Under heating in air, they withstand temperatures in excess of 900°C without any traces of structural degradation or chemical modification [7]. Also, due to a sort of ionic contribution into the B-N chemical bonding and preferential B-N-B-N stacking across the tubular multilayers, a BNNT has a characteristically straight shape (opposed to CNTs, which are usually waved, entangled, and curled) which makes it easy to achieve effective BNNT dispersion and/or texturing in any given matrix.

For more than a decade, our group has been working on such tubes and various composites made of them. Successfully fabricated polymer or ceramic-BNNT composites had indeed been reported [8-11]. Also, as the first try merging Al and BNNT functional properties, we succeeded in the fabrication of the so-called ‘Al-BNNTs nanocomposites’ using ion implantation and magnetron sputtering and carried out pioneering *in situ* tensile and bending tests on individual Al-BNNT composite structures in a high-resolution transmission electron microscope equipped with a piezo-driven manipulator [12]. As an outcome of these experiments, at least a nine-time increase in the tensile strength at room temperature was achieved on such nanocomposites compared to non-reinforced Al samples. The regarded nanomaterials consisted of a single BNNT core (20 to 50 nm in diameter) covered with a rather thick Al shield (up to 300 nm).

Thus, the next logic-driven step would be a try to design and to realize such lightweight BNNT-containing composites with meaningful dimensions (e.g., dozens of centimeter ranges) in which BNNTs are somehow distributed in a real crystalline Al matrix. As the initial step toward this goal, here, we report the first-time utilization of a melt-spinning technique to prepare BNNT-loaded lightweight Al composite ribbons.

## Methods

**Figure 1:**
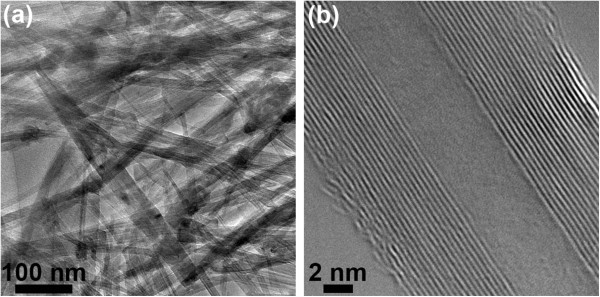
**TEM characterization of synthesized BNNTs.(a)** Representative low-magnification image of a BNNT ensemble. **(b)** High-resolution TEM image of an individual BNNT.

Multiwalled BNNTs were synthesized at a high yield (approximately 1 g per single experimental run) through the so-called boron oxide-assisted CVD (BOCVD) method, as was reported in our previous publications [9,10,12-14]. Figure 1 depicts low- and high-resolution TEM images of the prepared BNNTs. The length of BNNTs was 1 to 5 μm, and their average external diameter was 40 to 50 nm.

**Figure 2:**
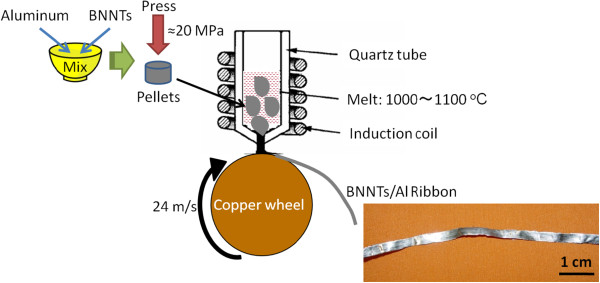
Fabrication procedure of Al-BNNT composite ribbons.

After subsequent high-temperature purification in argon atmosphere, the nanotubes were dispersed in ethanol. The Al-BNNT composites were cast using a melt-spinning technique in argon atmosphere. Figure 2 shows a sketch of the fabrication procedure.

To disperse BNNTs well within an Al powder, the tubes were kept in ethanol during their mixing with the powder (50 to 150 μm, purity 99.5%). It was a very important step as some tube clustering may occur under powder mixing and further formed Al-BNNT pellets cannot be electrically conductive (BNNT fraction is an electrical insulator). This would prevent their further melting under induction heating. The Al powder and dispersed BNNTs were mixed by a mechanical mixer; approximately 0.5 g of the mixed material was put in a die and pressed at a pressure of approximately 20 MPa at room temperature, and numerous starting Al-BNNT pellets were fabricated. Al-BNNT composite ribbons were prepared using melt spinning (a machine by NISSIN-GIKEN Corporation, Iruma, Japan) in an argon atmosphere. About 2 to 2.5 g of the prepared Al-BNNT pellets were used for a single experimental run. They were pre-placed in a quartz tube, which had a nozzle diameter of 1 mm, melted by the induction currents, and melt-spun on a rotating water-cooled copper drum at a wheel rotation speed of 24 m s^−1^. The fabricated melt-spun ribbons were approximately 50 μm in thickness and 4 to 5 mm in width. The length of the ribbons varied and was dependent on the stability of casting. As a rule, the fragments up to 1 m long could be obtained.

The phase compositions and crystal structures of the prepared composites were analyzed by X-ray diffraction (XRD; RINT2000 Ultima III, Rigaku Corporation, Tokyo, Japan) using Cu *K*α_1_ radiation. The morphologies and micro- and atomic structures of the composite ribbons were studied by scanning electron microscopy (SEM; S4800, Hitachi Ltd., Tokyo, Japan) and high-resolution transmission electron microscopy (TEM; 300 kV JEM-3000F, JEOL and JEM-3100FEF (Omega filter) instruments, JEOL Ltd., Akishima, Tokyo, Japan). TEM samples were prepared by using focused ion beam (FIB) polishing. Energy dispersive X-ray spectrometry under SEM and TEM investigations (EMAX EX-220, Horiba Ltd., Kyoto, Japan; JEM-3100FEF microscopes) at accelerating voltages of 10 kV (SEM) and 300 kV (TEM), respectively, were employed to identify the composite chemistry and to spatially map the constituting species. Tensile tests were carried out at room temperature on a ‘Shimadzu’ testing machine (AG-plus 10kN, SHIMADZU, Kyoto, Japan) at a deformation rate of 1.67 × 10^−4^ s^−1^.

## Results and discussion

**Figure 3:**
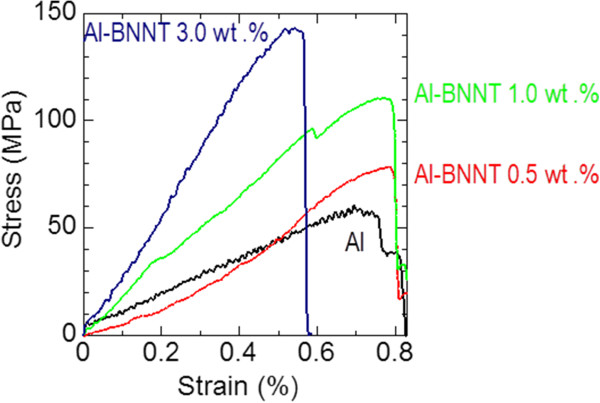
Stress–strain curves of pure Al and composite Al-BNNT melt-spun ribbons under tension at room temperature.

Representative room-temperature stress–strain curves of pure melt-spun Al ribbons and those with various BNNT loading fractions are shown in Figure 3.

The maximum measured strengths of ribbons are 60 MPa (Al), 75 MPa (Al-BNNT 0.5 wt.%), 115 MPa (Al-BNNT 1.0 wt.%), and 145 MPa (Al-BNNT 3.0 wt.%). The curves for Al and Al-BNNT 0.5 wt.% ribbons look nearly similar, meaning that at such low BNNT loading fractions, the tensile properties still cannot be modified. However, with increasing BNNT content, the tensile strength and the slope of the curves (and thus the Young’s modulus) dramatically change. For example, the ultimate tensile strength and the Young’s modulus more than doubled in the sample with 3 wt.% BNNTs.

It would be interesting to compare the obtained tensile strength data with the previously reported results for Al-CNT composites. For example, Kuzumaki et al. [15] measured these values for pure Al samples and for those with 2.5 and 5 wt.% of CNT loadings, before and after annealing the samples over 50 and 100 h at 873 K. The tensile strength values of 90 MPa for untreated Al samples, and 45 MPa and 40 MPa for these after consecutive annealing, and unchanged values of 80 MPa (either before or after heat treatments) for the samples with CNTs were reported. Salas et al. [16] documented only 20 MPa strength for Al samples with 5 wt.% of CNTs. Therefore, the figures obtained in our work markedly prevail over literature data for Al-CNT composites at approximately the same or even lower loading fractions of reinforcing BNNTs.

**Figure 4:**
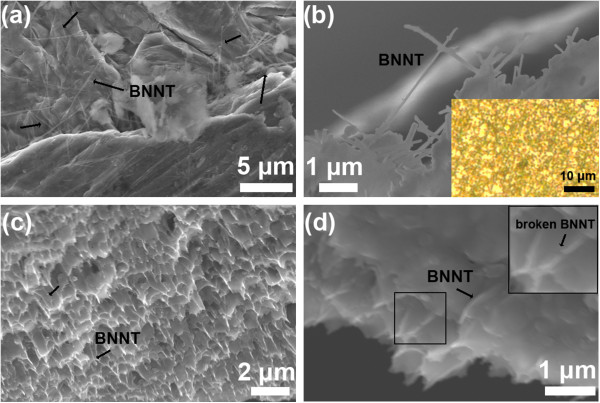
**Structural characterization of samples.(a)** SEM image of Al-BNNT (3 wt.%) composite pellet before melt casting. **(b)** A morphology formed in the melt cast ribbon; the inset in **(b)** is an optical image of the cast ribbon after polishing and etching; this reveals an approximately 1.5- to 3-μm Al grain size. **(c**, **d)** Representative fracture surfaces of the tensile-tested sample (3 wt.% BNNT) at various magnifications; individual BNNTs are seen at those surfaces (arrowed); thus they, at least partially, carry the applied tensile load and participated in the deformation process. A framed area shows a tube presumably broken into two halves under tension; this area is specially enlarged in the upper-right inset.

Figure 4a shows a SEM image taken from a starting Al-BNNT 3 wt.% pellet before melt casting. Individual (not bundled) BNNTs are seen randomly distributed within the pellet (as arrowed). The typical tube length reaches approximately 5 μm. Figure 4b depicts a SEM image of the same sample after melt casting and FIB treatment.

The BNNT network is clearly seen at the edge of the Al matrix. Many nanotubes protrude out of the polished Al phase, creating a sort of internal microframe. The inset to this figure displays an optical image of the same ribbon after polishing and chemical etching of its surface. Most of the Al grains after melt spinning are very fine, around only 2 to 3 μm in size. Figure 4c, d shows SEM images of the fractured surfaces of a Al-BNNT 3 wt.% ribbon after the tensile test. Some BNNTs embedded in the Al matrix are seen at that surface (arrowed), which is an indication of their contribution to carrying a tensile load.

The ribbon casting rate can hardly be controlled using the present experimental setup. It is determined by the specific melting conditions inside the inductor of the melt-spinning equipment, which sometimes may vary. Perfect texturing/orientation of BNNTs within the melt-spun ribbons is difficult to achieve, and the tubes are mostly randomly oriented within the ribbons, having only a sort of quasi-alignment along the casting direction. Viscosity of the melt can be controlled by its overheating; this may result in better nanotube orientation under casting, but the overheating can lead to the negative phenomenon of BNNTs floating to the upper molten zone and inhomogeneous tube distribution in the ribbons after casting.

**Figure 5:**
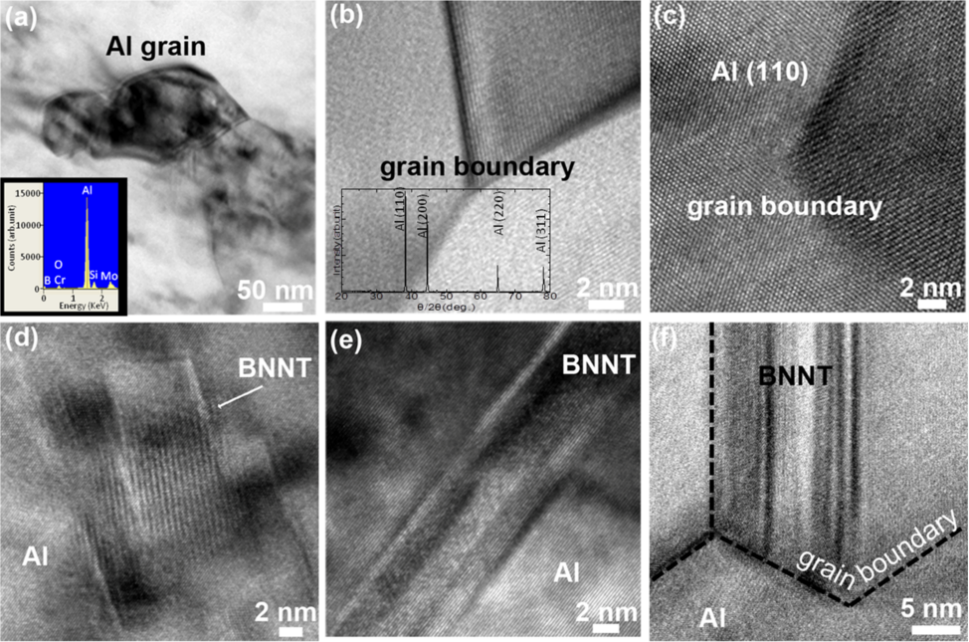
**TEM characterization of melt-spun ribbons.** TEM images of an Al-BNNT (3 wt.%) composite ribbon near the fractured surface after a tensile test. **(a)** The smallest Al grains found in the melt-spun Al-BNNT matrix; the inset depicts an EDS pattern recorded from this area. **(b**, **c)** A triple grain boundary in the Al-BNNT matrix at various magnifications; the central inset in **(b)** shows a representative X-ray spectrum confirming no other phases formed in the matrix except Al; the (110). (200), (220), and (311) Al peaks are marked. (**c**) In this case, the Al matrix is nicely oriented along the [110] zone axis of the fcc Al lattice. **(d** to **f)** A fading contrast peculiar to images relevant to individual multiwalled BN nanotubes present in the fractured ribbons either within the grains **(d** to **e)** or along the grain boundaries **(f)**.

Figure 5 shows low- and high-resolution TEM images, EDS, and XRD analyses of the obtained Al-BNNT 3 wt.% composite ribbons close to the fracture surfaces after the tensile tests. The EDS spectrum at the inset of Figure 5a confirms the pure Al composition of the matrix after melt casting - a weak B peak is coming out of the dispersed nanotubes, Cr and Mo peaks are due to the TEM holder, and minor Si and O signals are possibly originated from the traces of the quartz in the melt-spun samples. The clean Al micrograins and their triple boundaries are seen at a high magnification (Figure 5b); importantly, no other phases like Al borides or nitrides form in the Al matrix according to a detailed X-ray analysis on numerous samples (the central inset to Figure 5b depicts a representative X-ray spectrum).

The atomically resolved TEM image in Figure 5c displays a microcrystalline Al grain viewed along the [110] zone axis. The traces of remaining BNNTs embedded into the Al matrix are also apparent (Figure 5d, e, f). The nanotubes may be located inside the grains (Figure 5d, e) or be somehow assembled along the grain boundaries (Figure 5f).

The above-presented microscopic analysis revealed several important features of the nanotube-containing melt-spun material and its deformation process: (1) the multiwalled BNNTs are randomly distributed in the melt-spun ribbons; (2) no other phases except pure Al and well-preserved BNNTs are present in them; (3) BNNT cohesion strength with the metal is high enough and allows them not to be pulled out from the metal during tension; (4) the nanotubes, at least partially, carry the tensile load, as evidenced by their microscopic images for which the tube axes are somehow aligned along the deformation axis (for instance, Figure 4c, d), and sometimes the nanotubes are seen broken in pieces (the framed area and the corresponding inset) close to the fracture surface (Figure 4d).

It is known that nanophase precipitates can serve as pinning sites for the dislocation movement, thus leading to the Al matrix hardening. This mechanism has widely been accepted, and most likely, it is applicable here. In fact, the BNNTs distributed within or along the grain boundaries (Figure 5d, e, f) may hinder the dislocation glide and lead to the restriction of a plastic flow and matrix strengthening. Additionally, the particular appearance of nanotubes, which are seen being broken at the fractured surfaces (Figure 4d), tells us that a load transfer from the Al matrix to the reinforcing nanotubular agents has indeed taken place under room-temperature tension. The tensile strength of the reinforcing BNNTs is much higher compared to that of the pristine Al matrix (approximately 30 GPa [13,14] and 40 to 80 MPa, respectively); therefore, the former may effectively work during tension, if the nanotube orientation happens to be along the loading axis. More work is clearly needed to perfectly align the BNNTs and/or to texture them inside the Al matrix, and to check the deformation kinetics at the intermediate (100°C to 300°C) and high (400°C to 600°C) deformation temperatures. The effects of the Al grain growth and the influence of embedded BNNTs on this process should also be evaluated with respect to the mechanical properties at temperatures higher than the room temperature.

The room-temperature Young’s modulus determined from the slope of the curves in Figure 3 was increased under BNNT loading from approximately 15 GPa (for pure Al ribbons) to approximately 35 GPa (for the ribbons having 3 wt.% of BNNTs). It is noted that the determined Al ribbons’ Young’s modulus is several times lower compared to the literature data for the bulk Al. This may be caused by a microcrystalline nature of the samples and/or some morphological peculiarities of the presently cast ribbons, for instance, porosity. Therefore, the Young’s modulus of the present samples may only be compared qualitatively from sample to sample, rather than with other Al materials; taking this into account, one may document more than a two-time increase from pure Al to a composite ribbon with 3 wt.% of BNNTs. The obtained composite tensile strength values (maximum of 145 MPa) are much higher compared to pure Al (60 MPa). The analogous dramatic effects of multiwalled BNNTs on Al mechanical properties (under compression) were reported by Singhal et al. [17] who had used a powder metallurgy route and checked the microhardness and a compressive strength of the samples loaded with 1.5 wt.% BNNTs. These values were correspondingly increased five and three times compared to pure Al samples prepared under the same technology. It is worth noting that the present strength data for melt-spun Al-BNNT composite ribbons are comparable or somewhat lower than those for the cast or wrought Al alloys, for example, 483 MPa and 248 MPa for conventional 2014-T6 and 6063-T6 materials, and thus are still far from the satisfaction of engineers. But we believe that there is still a large room for improvement. The potential of BNNT strengthening has not been fully realized as yet due to several technological obstacles. The foreseen ways of the further Al-BNNT composite enhancement are proposed by us as follows: (1) increasing the BNNT loading fraction and the tube texturing/alignment in a given matrix, (2) functionalization and/or perforation of the external BNNT surfaces to increase their cohesion with the Al matrix, (3) pre-heat treatment of the ribbons before the tensile tests directed to the second phase precipitation at the BNNT-Al interfaces and increasing the efficiency of a load transfer via chemical bonding at the nanotube-metal interfaces, and (4) trying advanced powder metallurgy routes, i.e., spark-plasma sintering, to fabricate ultimately denser and larger BNNT-containing lightweight Al-based composites. Finally, it could be mentioned that combination of BNNTs and BN nanosheets [7] as a reinforcing phase in Al-based composites may also be an interesting direction. Such complex hybrids may possess an enhanced efficiency of the load transfer from a weak Al matrix to the strong and resilient nano-BN phases. These are the topics of our ongoing research.

## Conclusions

In summary, for the first time, we fabricated Al-BNNT composite ribbons (up to 1 m long) with various multiwalled BNNT contents (0.5 to 3.0 wt.%) by melt spinning. Scanning and transmission electron microscopy, X-ray diffraction, and energy dispersive X-ray analysis confirmed the decent integration of the two phases into a dense and compact composite. No other phases, like Al borides or nitrides, form in the resultant melt-spun composites. The BNNTs are randomly oriented within the Al matrix and partially participate in carrying the tensile load, as evidenced by their presence and breakage at the composite fracture surfaces. The ultimate tensile strength of the composite ribbons with 3 wt.% of BNNT at room temperature was more than doubled (145 MPa) compared to non-loaded pure Al ribbons (60 MPa).

## Competing interests

The authors declare that they have no competing interests.

## Authors’ contributions

YM made the composite ribbon samples and performed microscopic characterizations and tensile tests under the supervision of DG. AP took part in the SEM analysis of the fracture surfaces. YM, AP, and DG wrote the final manuscript. DMT, CZ, YB, KF, and DVS took part in the discussion of the results and read and approved the final manuscript. All authors read and approved the final manuscript.
